# Addressing model error through atmospheric stochastic physical parametrizations: impact on the coupled ECMWF seasonal forecasting system

**DOI:** 10.1098/rsta.2013.0290

**Published:** 2014-06-28

**Authors:** Antje Weisheimer, Susanna Corti, Tim Palmer, Frederic Vitart

**Affiliations:** 1 European Centre for Medium-Range Weather Forecasts (ECMWF), Reading, UK; 2 Department of Physics, National Centre for Atmospheric Science (NCAS), University of Oxford, Oxford OX1 3PU, UK; 3 Instituto di Scienze dell’Atmosfera e del Clima (SAC), Consiglio Nazionale delle Ricerche (CNR), Bologna, Italy

**Keywords:** atmospheric science, meteorology, complexity

## Abstract

The finite resolution of general circulation models of the coupled atmosphere–ocean system and the effects of sub-grid-scale variability present a major source of uncertainty in model simulations on all time scales. The European Centre for Medium-Range Weather Forecasts has been at the forefront of developing new approaches to account for these uncertainties. In particular, the stochastically perturbed physical tendency scheme and the stochastically perturbed backscatter algorithm for the atmosphere are now used routinely for global numerical weather prediction. The European Centre also performs long-range predictions of the coupled atmosphere–ocean climate system in operational forecast mode, and the latest seasonal forecasting system—System 4—has the stochastically perturbed tendency and backscatter schemes implemented in a similar way to that for the medium-range weather forecasts. Here, we present results of the impact of these schemes in System 4 by contrasting the operational performance on seasonal time scales during the retrospective forecast period 1981–2010 with comparable simulations that do not account for the representation of model uncertainty. We find that the stochastic tendency perturbation schemes helped to reduce excessively strong convective activity especially over the Maritime Continent and the tropical Western Pacific, leading to reduced biases of the outgoing longwave radiation (OLR), cloud cover, precipitation and near-surface winds. Positive impact was also found for the statistics of the Madden–Julian oscillation (MJO), showing an increase in the frequencies and amplitudes of MJO events. Further, the errors of El Niño southern oscillation forecasts become smaller, whereas increases in ensemble spread lead to a better calibrated system if the stochastic tendency is activated. The backscatter scheme has overall neutral impact. Finally, evidence for noise-activated regime transitions has been found in a cluster analysis of mid-latitude circulation regimes over the Pacific–North America region.

## 1. Introduction

This paper is a contribution to the *Philosophical Transactions of the Royal Society A* Theme Issue devoted to ‘Stochastic modelling and energy-efficient computing for weather and climate prediction’. It describes and discusses results presented by the authors at a workshop in Oxford, UK, in March 2013 (https://www.maths.ox.ac.uk/groups/occam/events/stochastic-climate). The Oxford workshop brought together meteorologists, physicists, mathematicians and computer scientists to report recent progress in the representation of inherent uncertainties in weather and climate models using stochastic approaches, and to discuss how innovative ideas of fast and energy-efficient approximate computing hardware could be used to improve simulations in probabilistic weather and climate models [1].

The motivation for including stochastic approaches in our current generation of weather and climate models is clearly set out in a recent essay by Palmer [2]: deterministic parametrizations in sophisticated weather and climate models are inconsistent with the implications of the scaling symmetries in the Navier–Stoke equations and the observed power-law behaviour in the atmosphere. These structures prevent a meaningful separation between resolved and unresolved scales. One important consequence of the power-law structure in the atmosphere is the upscale error propagation where errors at very small scales (very high horizontal model resolution) can grow and ultimately contaminate the accuracy of simulations at much larger scales in a finite time. Thus, any parametrization needs to take into account the representation of the sub-grid-scale model uncertainty on the resolved scales.

Successful applications of stochastic approaches in numerical weather prediction (NWP) have evolved in the recent decade from the early attempts in the European Centre for Medium-Range Weather Forecasts (ECMWF) ensemble prediction system [3] to operational schemes at several meteorological services around the world [4,5]. The currently operational medium-range ensemble prediction system at ECMWF includes two packages to represent model uncertainty, the stochastically perturbed physical tendencies (SPPT) scheme and the stochastically perturbed backscatter (SPBS) scheme. The SPPT scheme is based on the Buizza *et al.* [3] scheme and applies stochastic perturbations in the form of multiplicative noise to the diabatic (parametrized) part of the tendency equations of the prognostic variables [6]. The SPBS scheme aims at describing the mechanism of stochastic backscatter of kinetic energy from the near-truncation scales to the larger scales, as originally pioneered in large Eddy simulations [7]. Both schemes were shown to not only improve the quality of NWP forecasts, but also to reduce some systematic errors in these models [6].

Apart from NWP, stochastic parametrizations have not been widely applied in general circulation models of the atmosphere or the coupled climate system. As a step towards reducing this gap between the use of these new stochastic approaches in weather and climate simulations, this study presents an analysis of the impact of these techniques on ECMWF's operational long-range predictions on seasonal time scales. Unlike conventional weather forecasts, seasonal predictions do not attempt to forecast the detailed day-to-day evolution of weather because of the chaotic nature of the climate system. Rather, seasonal predictions provide estimates of forecast seasonal means of the coming season. The physical basis for such estimates arises from the effect of predictable seasonal time-scale signals arising from the ocean, and to a lesser extent the land surface, on the atmosphere [8]. The key paradigm for seasonal forecasting is the El Niño southern oscillation (ENSO), a coupled ocean–atmosphere phenomenon occurring primarily in the tropical Pacific and predictable six months and more ahead [9,10].

ECMWF has been at the forefront of seasonal predictions for many years. Research on predictability on seasonal time scale in the early 1990s led to the implementation of the first ECMWF seasonal forecast system based on a global ocean–atmosphere coupled model in 1997, and a successful forecast of the major 1997–1998 El Niño [11]. The first coupled seasonal forecast System 1 was replaced by System 2 in 2001 and System 3 in March 2007. In November 2011, the latest seasonal forecasting system—System 4—started producing operational forecasts. As System 4 uses for its atmospheric component a version of ECMWF's model for NWP that includes the latest development of the SPPT and SPBS stochastic parametrization schemes, it provides an ideal test bed for studying the impact of the perturbed tendency and backscatter scheme on seasonal climate forecasts. Positive results found in previous investigations with earlier non-operational versions of the ECMWF model using a cellular automaton pattern for the backscatter scheme [12] and comparing the stochastic model error approach with multi-model and perturbed physical parameter ensemble methodologies [13] motivated the research described here. The results presented in this paper are based on System 4's retrospective seasonal forecasts by comparing them with a set of simulations without stochastic representations of model uncertainty.

Based on studies using simplified climate models with stochastic noise, it was found that these perturbations can impact on the mean state of the system through noise-induced drift [14,15] and trigger noise-activated regime transitions [16]. Suppose the system has a potential well structure with double minima, which would correspond to a bimodal non-Gaussian probability density function (pdf). While statistically the most frequent state of the system would be the one that corresponds to the absolute potential well minimum and the absolute maximum of the pdf, the system would also, though less frequently, visit the other quasi-stable mode if the forcing applied is strong enough to allow for it. Suppose a small stochastic forcing is now applied to the system. While most of the time it might be too weak to move the system out of its preferred state, some perturbations might be large enough to kick the system more often than normal to the other less frequent mode of variability. In a statistical sense, these noise-activated regime transitions will lead to changes in the mean state of the system and in the frequency of occurrence of the two modes of variability of the system.

In this study, we analyse the impact of stochastic perturbations on the long-term statistics and biases in the ECMWF coupled seasonal forecasting model. We find evidence for both noise-induced drift of the large tropical convective areas and noise-activated transitions between the preferred atmospheric circulation regimes over the extratropical North Pacific–North America region. The paper is structured as follows. Section 2 describes the seasonal forecasting System 4 that is used to study the impact of stochastic parametrizations. Section 3 discusses results of the change in the mean state by looking at the individual contributions of the SPPT and SPBS schemes. The impact of these two schemes on the dominant tropical mode of intraseasonal variability, the Madden–Julian Oscillation (MJO), is the subject of §4. Tropical Pacific sea-surface temperature (SST) forecasts of ENSO are discussed in §5, whereas §6 shows how the impact of stochastic perturbations manifests itself in a cluster analysis of quasi-stationary circulation regimes over the Pacific–North America (PNA) region. A summary of the main results and some conclusions are presented in §7.

## 2. ECMWF's seasonal forecasting System 4

ECMWF's seasonal forecasting System 4 [17] consists of an atmospheric and an oceanic model component to simulate the evolution of the global circulation in the atmosphere and in the oceans, based on the physical laws of fluid dynamics. The equations of motion and thermodynamics are solved numerically by discretizing the atmosphere and the oceans vertically and horizontally. The atmospheric component of System 4 is version CY36R4 of ECMWF's state-of-the-art NWP model Integrated Forecast System (IFS). While the dynamical part of the model solves the equation of motion for adiabatic processes, many physical processes, for example those related to phase changes of water and the formation of convection and clouds, operate on sub-grid scales and thus cannot be resolved explicitly by the finite coarse resolution of the atmospheric model grid. In the IFS model, these unresolved diabatic processes are described through a set of deterministic physical parametrization schemes.

As mentioned in the Introduction, two approaches to represent uncertainties in these physical parametrizations have recently been included in the IFS [6] in the form of stochastic physical parametrization schemes. In the SPPT scheme, the summed tendencies of the prognostic variables temperature, wind and humidity as passed on from the individual parametrization schemes are perturbed with a multiplicative univariate Gaussian noise term. The perturbations vary smoothly following an autoregressive model of order 1 (AR1) process in space and time with three distinct spatio-temporal scales with characteristic lengths of 500, 1000 and 2000 km. The corresponding temporal scales (*e*-folding times) are 3 h, 3 and 30 days. The shortest scale is connected with the largest amplitude of the perturbations, whereas the longest and slowest scale becomes active via small perturbations. The choice of the amplitude of the perturbations has been motivated by results from coarse-graining studies with cloud-resolving models [6,18,19]. The parameter settings for SPPT used in System 4 are exactly the same as those used in ECMWF's operational medium-range weather forecasting system.

The second scheme, the SPBS scheme, describes a physical process missing in conventional parametrization schemes: the upscale energy transfer from unbalanced motions associated with convection and gravity waves and from the balanced flow in the manner of two-dimensional (or quasi-geostropic) turbulence. It is formulated in terms of a perturbed streamfunction forcing whose amplitude is modulated by the total dissipation rate (sum of numerical dissipation, dissipation owing to orographic gravity wave drag and that owing to convection). The backscatter ratio, which determines the energy input rate in the streamfunction forcing, depends on the horizontal model resolution and is set to 0.04 in the System 4 configuration. For detailed descriptions of these two schemes, see Palmer *et al.* [6], Shutts [20], Berner *et al.* [7] and Shutts & Callado Pallarés [19].

The atmospheric model IFS is run in horizontal spectral resolution T255, corresponding to a grid size of approx. 80 km, and 91 vertical levels up to 0.01 hPa. The ocean model used in System 4 is the Nucleus for European Modelling of the Ocean (NEMO) version 3.0, a state-of-the-art three-dimensional general circulation model. The ocean model has 42 levels in the vertical and the grid boxes have an approx. length of 110 km (1^°^) with equatorial refinement. The atmosphere and the ocean are coupled using the OASIS3 coupler to interpolate between the oceanic and atmospheric grids with a coupling interval of 3 h.

In order to achieve a robust estimate of the System 4 model performance, an extensive set of retrospective forecasts (re-forecasts) of the past has been generated and contrasted with verification data over that period. These re-forecasts form the basis of the analysis presented here. The System 4 re-forecasts were started every calendar month over the 30 year period 1981–2010 by emulating real forecast conditions when no observed information about the future is available at the beginning of the forecast. As discussed in the Introduction, seasonal forecasts must be probabilistic by nature. Thus, the seasonal forecasts and re-forecasts produced by System 4 consist of ensembles of model integrations. For the operational forecasts, each month a forecast ensemble of 51 members is started using slightly different initial conditions in the atmosphere and ocean. Owing to computational constraints, the default ensemble size of the re-forecasts is currently 15. However, for a subset of selected start months, the re-forecast ensemble size was extended so that it matches the forecast ensemble size of 51. These extended hindcasts are available for February, May, August and November start dates over the 1981–2010 period.

The ensemble of initial conditions in the re-forecasts is generated through a combination of ocean and atmospheric re-analyses and perturbations to the surface wind and SST fields over the ocean [17]. Here, the ORA-S4 NEMOVAR reanalysis [21] provides five equally likely realizations of the three-dimensional oceanic state at any given time during the re-forecast period. ORA-S4 has been used to initialize the ocean in the System 4 hind- and forecasts. The atmosphere in the re-forecasts is initialized using the ERA-Interim reanalysis [22] and ECMWF's operational configuration of singular vectors in the atmosphere. Each member of the ensemble has a different realization of the stochastic representation of the sub-grid-scale physical processes in the atmosphere using the SPPT and SPBS schemes.

Here, we analyse 51 member ensemble re-forecasts initialized on 1 May and 1 November 1981–2010. The standard forecast lead time is two to four months, corresponding to the boreal summer (June, July and August; JJA) and winter (December, January and February; DJF) seasons. For the MJO analysis in §4 forecast lead times of up to six months were considered and the re-forecasts initialized on 1 August 1981–2010 have also been used.

To distinguish clearly the impact of the stochastic physical parametrization schemes on System 4, several control experiments have been performed with an identical set-up to that in System 4 with stochastic schemes switched off either separately or simultaneously. In the *stochphysOFF* experiment, both the SPPT and the SPBS schemes were switched off. The experiment labelled *SPPT_ON* has the SPPT scheme activated but not the SPBS scheme. Similarly, the experiment labelled *SPBS_ON* only uses the SPBS scheme, whereas the SPPT scheme is switched off.

## 3. Stochastic physical parametrizations and the mean state of the atmosphere

Here, we describe the impact of the two stochastic physical parametrziation schemes SPPT and SPBS on the mean state and systematic errors of the atmosphere in System 4. Stochastic perturbations have, through a process called noise-induced drift, the potential to change the mean state of a system [14] and [15]. While the overall impact of the stochastic physics schemes on the mean atmospheric state is small, we find that the perturbed tendency scheme SPPT does have an impact in the convectively dominated tropical areas where it reduces some of the systematic errors of the coupled ECMWF system.

**Figure 1.**
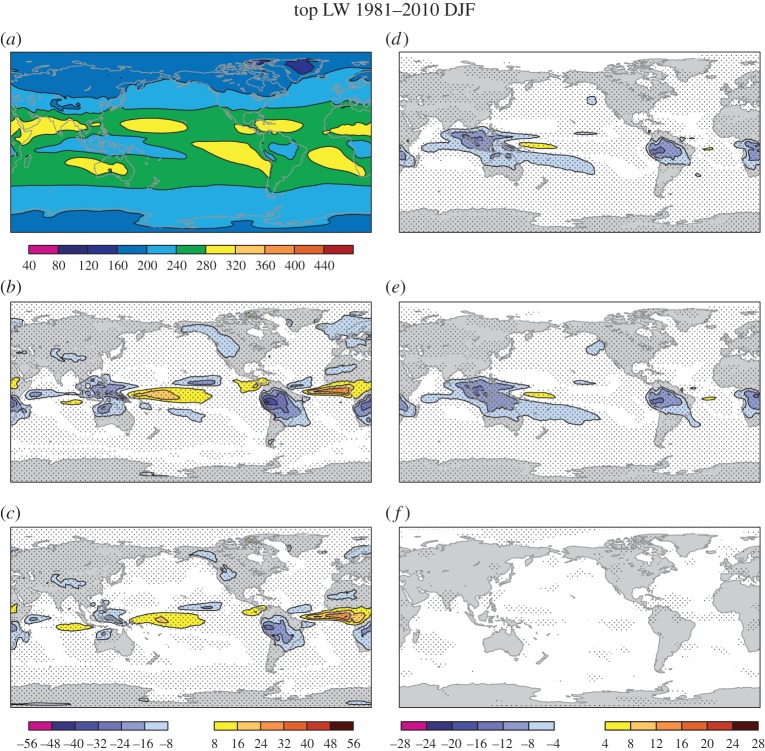
Top of the atmosphere net longwave radiation (outgoing longwave radiation; OLR) in W m^−2^ in DJF. (*a*) ERA-Interim reanalysis, (*b*) *stochphysOFF*−reanalysis, (*c*) System 4−reanalysis, (*d*) *stochphysOFF*−System 4, (*e*) *stochphysOFF*−*SPPT_ON*, (*f*) *stochphysOFF*−*SPBS_ON*. Significant differences at the 95% confidence level based on a two-sided *t*-test are hatched.

Figure 1*a* shows the net longwave radiation at the top of the atmosphere (OLR) in DJF as a proxy for convection as estimated in ERA-Interim over the hindcast period 1981–2010. The areas of large convection over the Indonesian warm pool area, South America and Africa show relatively small values of OLR corresponding to relatively cold temperatures over these convectively dominated regions. The bias of the control experiment *stochphysOFF* (figure 1*b*) based on November start dates of the re-forecasts during the same period indicates that these are also the areas where the model underestimates the fluxes owing to excessively strong convective activity. The biases are notably reduced in System 4 (figure 1*c*), where the stochastic physical parametrization schemes are activated. The difference between *stochphysOFF* and System 4 is displayed in figure 1*d*. It is the perturbed tendency scheme SPPT that leads to the reduced bias, as shown in figure 1*e*,*f* for the difference between the control experiments *stochphysOFF* and *SPPT_ON* or *SPBS_ON*.

**Figure 2.**
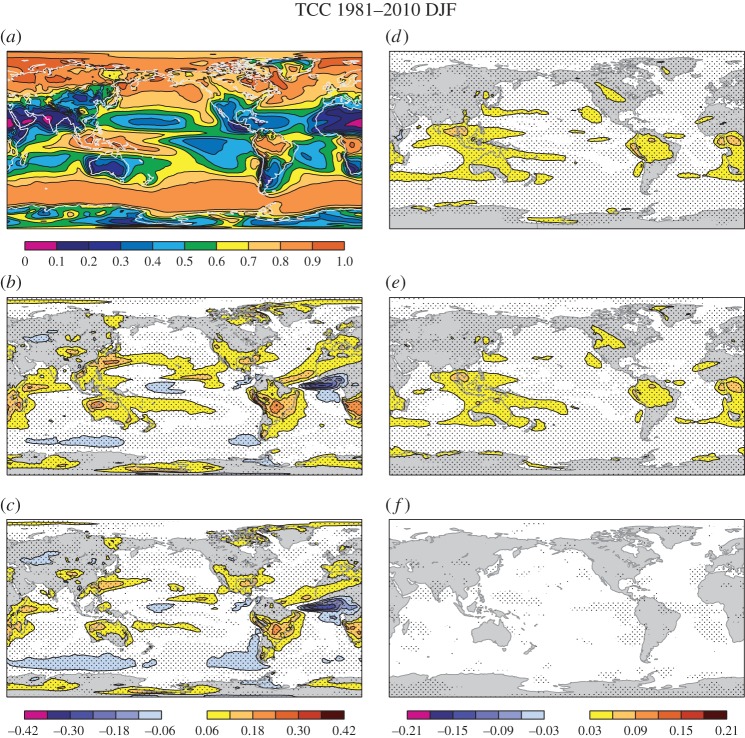
Total cloud cover in DJF. (*a*) ERA-Interim reanalysis, (*b*) *stochphysOFF*−reanalysis, (*c*) System 4−reanalysis, (*d*) *stochphysOFF*−System 4, (*e*) *stochphysOFF*−*SPPT_ON*, (*f*) *stochphysOFF*−*SPBS_ON*. Significant differences at the 95% confidence level based on a two-sided *t*-test are hatched.

Similar conclusions can be drawn from the diagnostics of the total cloud cover in DJF over the three main tropical convection areas (figure 2). The SPPT scheme (figure 2*e*) reduces the systematic overestimation of cloud cover in the *stochphysOFF* control experiment (figure 2*b*), whereas the SPBS scheme (figure 2*f*) has no impact on the cloud cover. The overall combined effect of SPPT and SPBS is an improved mean cloud cover when compared with ERA-Interim (figure 2*c*).

**Figure 3.**
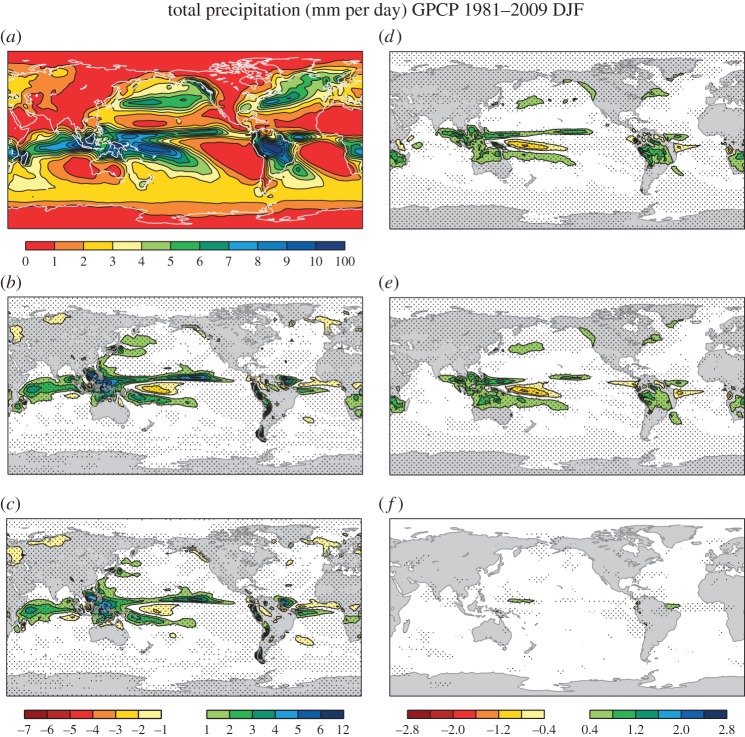
Total precipitation in mm per day in DJF. (*a*) GPCP, (*b*) *stochphysOFF*−GPCP, (*c*) System 4−GPCP, (*d*) *stochphysOFF*−System 4, (*e*) *stochphysOFF*−*SPPT_ON*, (*f*) *stochphysOFF*−*SPBS_ON*. Significant differences at the 95% confidence level based on a two-sided *t*-test are hatched.

Convective activity is strongly coupled to precipitation, and it is thus expected that the stochastic physical parametrization schemes impact on the precipitation fields. Figure 3 demonstrates that the model in the control experiment *stochphysOFF* (figure 3*b*) produces excessive amounts of tropical precipitation in DJF, in particular, over the warm pool and tropical Western Pacific. Here, the verification is done against Global Precipitation Climatology Project (GPCP) precipitation data [23,24], because the reanalysis estimates for tropical precipitation in ERA-Interim have non-negligible deficiencies [22]. As for the longwave radiation at the top of the atmosphere and total cloud cover, the SPPT scheme (figure 3*e*) contributes the most to an overall reduction of that positive precipitation bias in System 4 (figure 3*c*,*d*).

**Figure 4.**
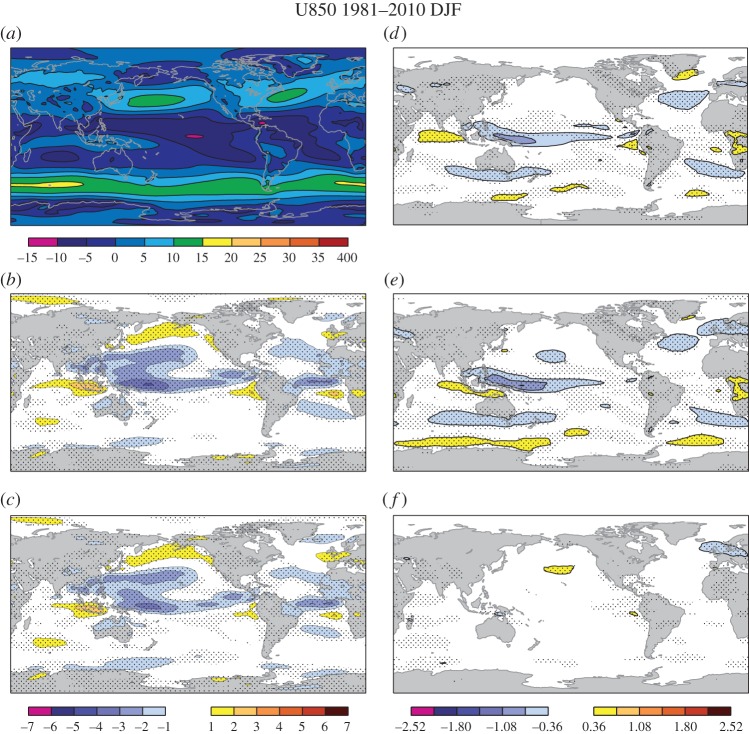
Zonal wind at 850 hPa in m s^−1^ in DJF. (*a*) ERA-Interim reanalysis, (*b*) *stochphysOFF*−reanalysis, (*c*) System 4−reanalysis, (*d*) *stochphysOFF*−System 4, (*e*) *stochphysOFF*−*SPPT_ON*, (*f*) *stochphysOFF*−*SPBS_ON*. Significant differences at the 95% confidence level based on a two-sided *t*-test are hatched.

The stochastic physical parametrization schemes, and in particular SPPT, also have a positive impact on the seasonal-mean zonal wind climatology at 850 hPa in the tropics (figure 4). In the control experiment *stochphysOFF* without any representation of model error through stochastic parametrizations, the model has also strong easterly trade winds in the equatorial regions. This is especially true for the tropical Western and Central Pacific. This overestimation of the easterlies is reduced when the stochastic physics schemes are implemented in System 4 (figure 4*c*). Again, the SPPT scheme is the main reason for the improvement with SPBS having a nearly neutral impact.

Summarizing the findings discussed above, we conclude that the stochastic physical parametrizations have an overall positive impact on the DJF climatology of atmospheric fields (OLR, cloud cover, precipitation and near-surface wind) relevant for the large convectively active regions over the Indonesian warm pool/tropical Western Pacific area as well as over tropical South America and Africa. While the amplitude of the long-standing systematic errors for these quantities was not eliminated completely, the results from the stochastic schemes show a consistent improvement with a spatial projection onto the pattern of the coupled model bias. The reported model biases are already detectable during the first month of the simulations with a similar pattern and amplitude (not shown). Even though the emphasis in this section was on the DJF season, qualitatively similar conclusions hold also for the JJA season.

## 4. Impact on the statistics of the Madden–Julian oscillation

The MJO is the dominant mode of intraseasonal variability in the tropical atmosphere [25,26] linking the large-scale atmospheric circulation with organized tropical convection on a range of spatial scales such as mesoscale convective systems and convectively coupled waves. It is characterized by an eastward propagation of areas of enhanced and suppressed tropical rainfall mainly over the Indian and Pacific Oceans with a time scale of 30–60 days. The MJO has important links to the developments of the monsoon systems and El Niño events in the tropical Pacific [27] as well as to extratropical predictability in the mid-latitudes [28,29].

Despite recent progress, understanding the dynamical mechanisms that lead to MJO events and realistically simulating the MJO in atmospheric circulation models remains a challenge for observational activities and modelling studies. New approaches such as superparametrization [30], multi-cloud [31–33] and multi-scale models [34] are showing a promising capability to improve the simulation of the interaction between organized tropical convective processes. Another route of current research is the investigation of how air–sea coupling affects the MJO [35,36].

Thual *et al.* [37] have suggested that stochastic parametrizations of unresolved synoptic processes in a minimal dynamical skeleton model of the MJO can account for some of the characteristic features of the MJO such as the intermittent generation of MJO events and the organization of MJO events into wave trains with growth and demise. While the role of stochasticity in forecasting the MJO using nonlinear empirical prediction methods has recently been demonstrated by Kondrashov *et al.* [38], it is not clear whether comprehensive general circulation models with stochastic parametrizations for the sub-grid-scale (convective) variability can improve the representation of the MJO in such complex models.

Here, we analyse the impact of the SPPT and SPBS stochastic parametrization schemes on the statistics of simulating MJO events in the System 4 re-forecasts. As the current predictability limit of the MJO in a recent version of the coupled ECMWF monthly forecasting system has been estimated to not reach more than 30 days [39,40], we cannot expect skilful MJO forecasts beyond one month for individual events in the seasonal simulations. However, we can test how well the MJO is represented in System 4 from a statistical point of view where we do not attempt to match individual MJO events in the reanalysis with events in the seasonal forecasts. Our focus here is on the frequency of occurrence of MJO events in the different stages of its propagation from West to East and on the amplitude distribution of the MJO events under the impact of the stochastic perturbation schemes.

**Figure 5.**
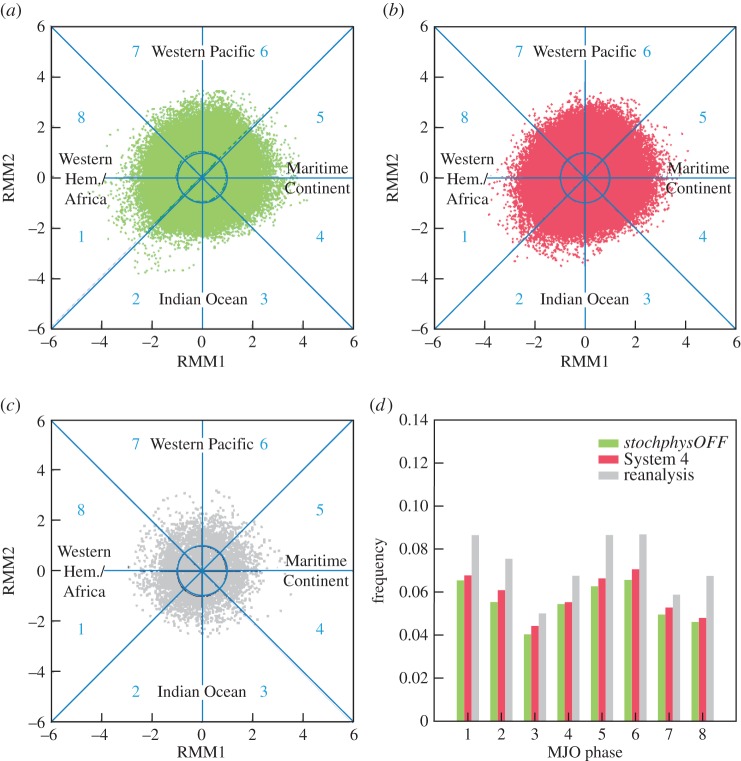
MJO frequencies. (*a*) Wheeler–Hendon diagram for *stochphysOFF*. The dots show daily data over the 1981–2010 hindcast period for May start dates, forecast lead times up to six months and all ensemble members. (*b*) As in (*a*) but for System 4. (*c*) Similar to (*a*) but for ERA-Interim reanalysis. (*d*) Relative frequencies of MJO events in each of the eight MJO phases for the data in (*a*–*c*).

The methodology for assessing the MJO follows Gottschalck *et al.* [41]. The Wheeler and Hendon index (WHI; see [42]) has been applied to all model forecasts and to ERA-Interim to evaluate how well the seasonal forecasting system can reproduce the distribution of MJO events. The multi-variate WHI is calculated by projecting the forecasts or analysis on the two dominant combined empirical orthogonal functions (EOFs) of OLR, zonal wind at 200 and at 850 hPa averaged over the near-equatorial latitudes 15^°^N and 15^°^S. The index has been applied to daily anomalies relative to model climatology instead of the absolute value of the field, in order to remove the impact of the annual cycle. In addition, a 120 day running mean has been subtracted to remove the variability associated with ENSO. The positive (negative) phase of EOF 1 describes enhanced (suppressed) convection over the Maritime Continent region. The positive (negative) phase of EOF 2 describes suppressed (enhanced) convection over the Indian Ocean and enhanced (suppressed) convection over the West Pacific. Analysis and forecasts are projected onto the two EOFs to describe the phase of the MJO in terms of two time series, the real-time multi-variate MJO series 1 (RMM1) and 2 (RMM2). The two time-series can be plotted as a succession of points in the RMM1–RMM2 phase space (called a Wheeler–Hendon diagram), in such a way that an evolving MJO event is described by a clockwise propagation in the phase space. The RMM1–RMM2 phase space can be divided into eight sections representing a specific phase of the MJO (figure 5*a*–*c*). Phases 2 and 3 (negative EOF 2) correspond to enhanced convection over the Indian Ocean, phases 4 and 5 (positive EOF 1) correspond to the MJO over the Maritime Continent, phases 6 and 7 (positive EOF 2) correspond to the MJO over the Western Pacific and phases 8 and 1 (negative EOF 1) correspond to the active phase of the MJO in the Western Hemisphere. Because of the loss of predictability of individual MJO events on seasonal time scales, we use the Wheeler–Hendon diagrams here to estimate the overall frequency of MJO events in each phase over the 30 year re-forecast period 1981–2010 for System 4, *stochphysOFF* and ERA-Interim.

The Wheeler–Hendon diagrams of the model runs in figure 5 show daily data of the re-forecasts starting in May and ranging for six months over the 30 re-forecast years and for all 51 ensemble members in the two-dimensional phase space defined by RMM1 and RMM2. The diagram in figure 5*a* displays the data for the control experiment *stochphysOFF* and in figure 5*b* for System 4. The corresponding data from the ERA-Interim reanalysis are shown for verification in figure 5*c*. Data points outside the unit circle are defined as MJO events where the amplitude of an MJO event is given by its distance from the origin of the diagram. Figure 5*d* summarizes the relative frequencies of MJO events (amplitudes greater than 1) in the eight MJO phases for the control *stochphysOFF* experiment, for System 4 and for ERA-Interim as estimated from counting all data points in the Wheeler–Hendon diagrams. The ECMWF model has a pronounced bias in underestimating the frequency of MJO events for all phases. However, the stochastic physical parametrizations used in System 4 increase the frequency of MJO events in each phase and thus reduce the negative bias. It is found (not shown) that this effect of the stochastic schemes increases with lead time from relatively little influence in the first month to a more pronounced impact at month 6. While the overall systematic underestimation of the number of MJO events in System 4 remains a problem, the stochastic schemes show a consistent positive but small impact. These results were confirmed for the two other available start dates in August and November.

**Figure 6.**
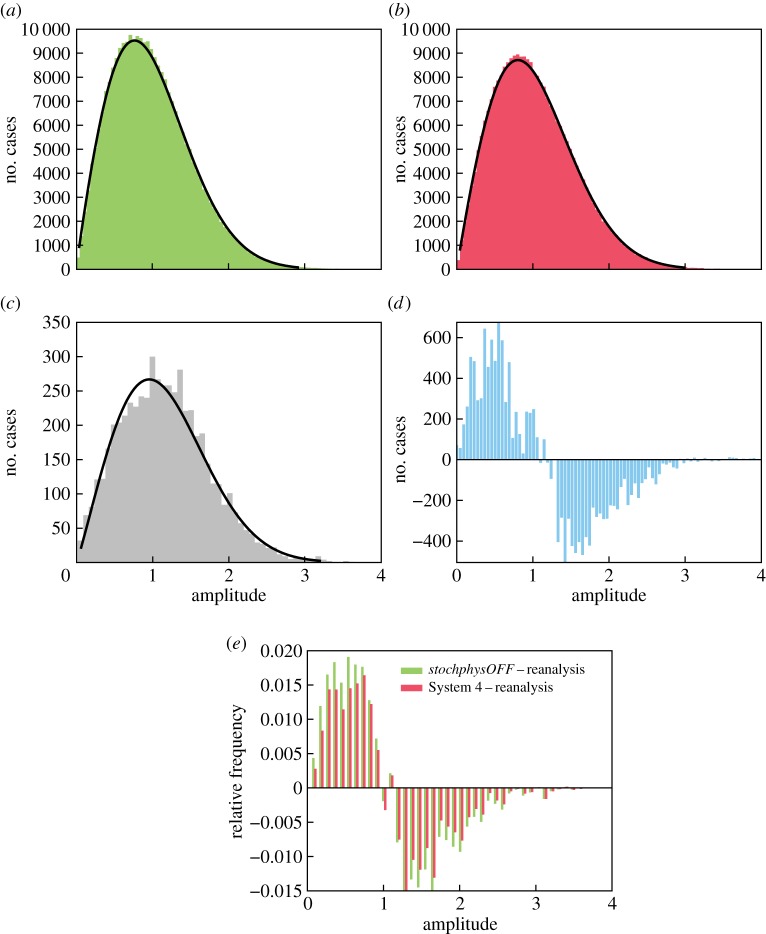
MJO amplitudes. (*a*) Distribution of the MJO amplitudes from the Wheeler–Hendon diagram in figure 5*a* for *stochphysOFF*. The fitted curve indicates a Weibull distribution. (*b*) As in (*a*) but for System 4. (*c*) Similar to (*a*) but for ERA-Interim reanalysis. (*d*) Residual distribution for *stochphysOFF*−System 4. (*e*) Residual distributions for *stochphysOFF*−reanalysis (green) and System 4−reanalysis (red).

In order to answer the question of what the impact of stochastic physics is on MJO events with different amplitudes, figure 6 quantifies the amplitude distributions for the *stochphysOFF* and System 4 re-forecasts and compares them with the distribution of the ERA-Interim verification data. The model histograms in figure 6*a*,*b* are much smoother than the reanalysis histogram in figure 6*c* because of the larger overall sample size using 51 ensemble members. The data in all three histograms show a good fit to a Weibull distribution. A Kolmogorov–Smirnov test indicates that the three distributions are significantly (*p*-values<0.01) different from each other.

The difference between the distributions in figure 6*a*,*b* is shown in the residual distribution in figure 6*d*. The seasonal re-forecasts without any representation of model uncertainty (*stochphysOFF*) generate more events with amplitudes smaller than 1 (not classified as MJO events). The impact of the stochastic physical parametrization schemes manifests itself in an increase in the number of stronger MJO events with amplitudes larger than 1.

This increase in the frequency of large-amplitude MJO events is an improvement in the model statistics of the MJO amplitude distribution. As shown in figure 6*e*, the frequency bias of System 4 estimated as the binned difference between the histograms of ERA-Interim and System 4 is for all amplitudes smaller than the frequency bias of *stochphysOFF*. It is clear that the stochastic physical parametrizations cannot eliminate completely the underestimation of MJO events when compared with ERA-Interim. However, the effect of introducing stochasticity reduces the underestimation consistently across the range of amplitudes.

**Figure 7.**
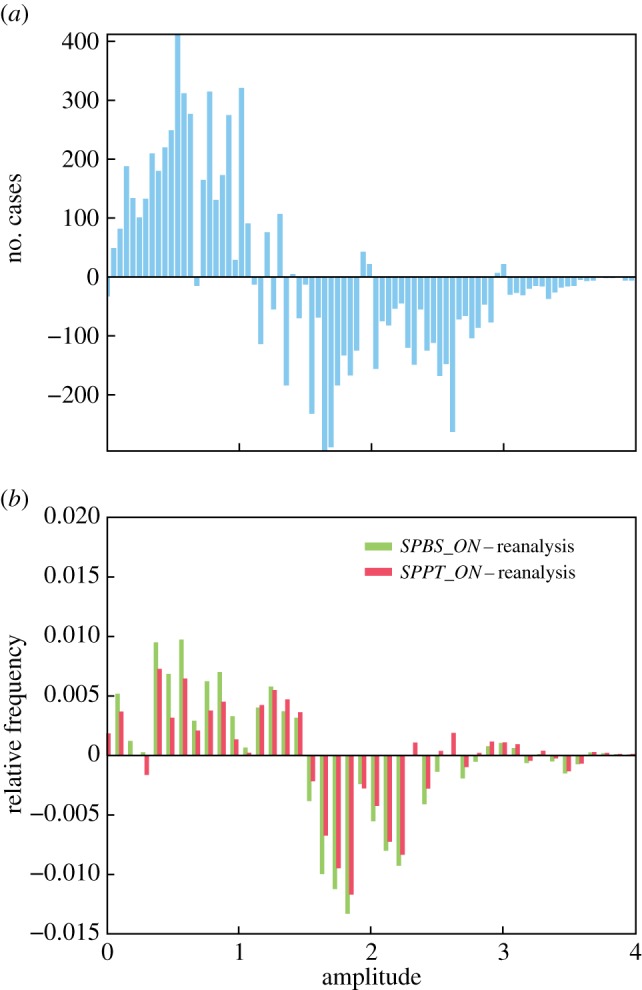
Impact of SPPT and SPBS on MJO amplitudes. (*a*) Residual distribution for *SPBS_ON*−*SPPT_ON*. (*b*) Residual distributions for *SPBS_ON*−reanalysis (green) and *SPPT_ON*−reanalysis (red).

What is the relative role of SPPT versus SPBS in the increased MJO activity? Figure 7*a* shows the residual distribution of MJO amplitudes between the SPBS_ON and SPPT_ON re-forecasts. Note that the *SPPT_ON* and *SPBS_ON* experiments have only been performed for November start dates and lead times of up to four months. The *SPBS_ON* experiment generates more events with amplitudes less than 1 (not classified as MJO events), whereas the *SPPT_ON* experiment increases the number of stronger amplitude MJO events. A comparison of the biases of the two experiments depending on amplitude (figure 7*b*) indicates that the *SPPT_ON* experiment develops for most cases a smaller bias (overestimation of weak events and underestimation of stronger events) than the *SPBS_ON* experiment.

## 5. El Niño forecast quality

As demonstrated in §3, the stochastic parametrization schemes in System 4 reduce some of the systematic errors of the coupled ECMWF forecasting system. In particular, the perturbed tendency scheme SPPT had a positive impact over the Indonesian warm pool area and the tropical Western Pacific by weakening the convective activity, reducing excessive precipitation and decreasing the strength of the excessively strong near-equatorial winds in the Western and Central Pacific. Here, we analyse how the representation of model uncertainty in System 4 and the related improvements of some of the systematic biases in these regions affect the forecast quality of ENSO on seasonal time scales.

**Figure 8.**
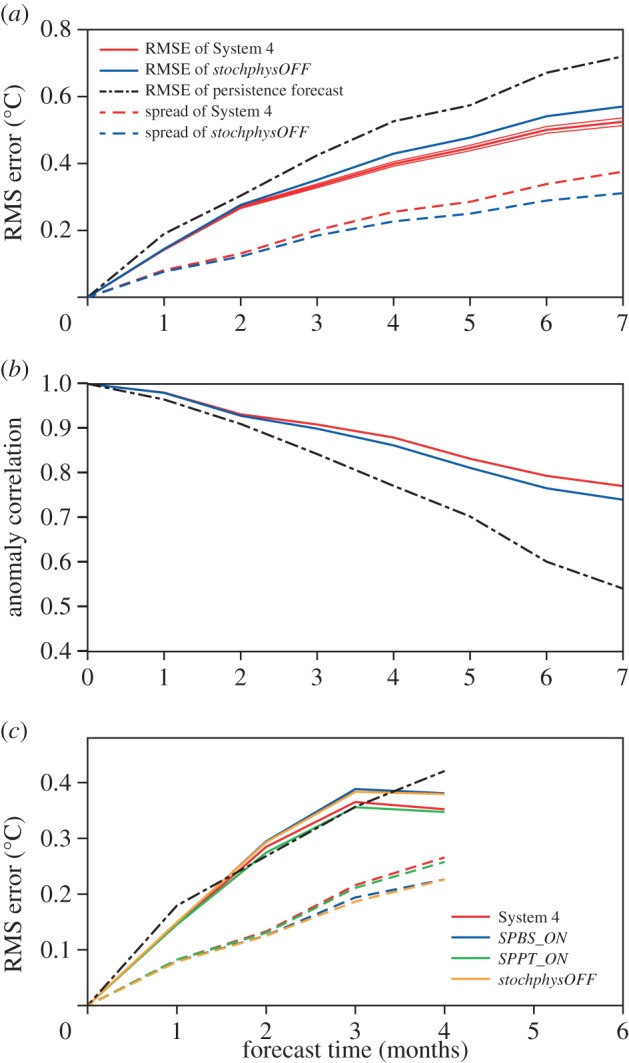
Forecast quality of SSTs in the Niño4 region. (*a*) RMSE of ensemble mean (solid lines), ensemble spread (dashed line) and RMS of persistence (black dashed-dotted line) as a function of forecast lead time for *stochphysOFF* (blue) and System 4 (red). Thin red lines indicate error bars of System 4 RMSE. Hindcast period: 1981–2010 May, August and November start dates. (*b*) Anomaly correlation for *stochphysOFF* (blue), System 4 (red) and persistence (black). (*c*) As in (*a*) but for System 4 (red), *SPPT_ON* (green), *SPBS_ON* (blue) and *stochphysOFF* (orange). Hindcast period: 1981–2010 November start dates only.

In figure 8*a*, we show the evolution over forecast lead time up to seven months of the root-mean squared error (RMSE) of the ensemble mean and ensemble spread (standard deviation) for SST forecasts in the tropical Western Pacific Niño4 area (160^°^E–150^°^W, 5^°^S–5^°^N) estimated from all available start dates 1 May, August and November over the re-forecast period 1981–2010. The solid red and blue lines show the RMSE, and the dashed red and blue lines below the ensemble spread. The RMSE of a simple statistical persistence forecast is shown for comparison as the black dash-dotted line above the other lines. All red lines correspond to the System 4 hindcasts, whereas the blue lines are from the experiment without stochastic parametrizations of model uncertainty *stochphysOFF*. The thin red lines around the RMSE of System 4 indicate the sampling uncertainty in estimating the RMSE. As can be seen, System 4 has a significantly lower RMSE than *stochphysOFF* for all lead times beyond two months. The improvement of System 4 over *stochphysOFF* becomes larger for longer lead times. The spread in the System 4 forecast ensemble is increased, which together with the reduced RMSE leads to a better calibrated forecasting system. Qualitatively similar results were found when analysing individual start dates and for the SSTs in other parts of the equatorial Pacific.

The anomaly correlation coefficient for the Niño4 SSTs over lead time is shown in figure 8*b*. Consistent with the findings for the RMSE, the correlation is improved when the stochastic physical parametrization schemes are activated in System 4.

For a subset of the re-forecasts (November start dates only over 1981–2010 for lead times up to four months) the individual contributions of the SPPT and SPBS stochastic perturbation schemes to the System 4 performance were tested (figure 8*c*). Here, the red lines correspond to System 4, the green lines to the *SPPT_ON* experiment with only the stochastically perturbed physical tendency scheme being active, the blues line to the *SPBS_ON* experiment where only the backscatter scheme was activated and the orange lines showing the results from the *stochphysOFF experiment*with no representation of model uncertainty. It can be seen that the System 4 and *SPPT_ON* re-forecasts are very similar in their magnitude of RMSE and spread evolution. By contrast, the *SPBS_ON* forecasts are very close to the control experiment *stochphysOFF*, in terms of both spread and error. This means that it is primarily the perturbed tendency scheme that generates a smaller RMSE and a larger spread of the ensemble compared with the control forecasts. The backscatter scheme by itself has only a very small impact.

The improvements owing to the stochastic parametrizations in System 4 in reducing both the RMSE and under-dispersiveness of ENSO forecasts may appear small. However, they are approximately of the same order as the effects of changing the physical parametrization packages and model configurations as occurs from one ECMWF seasonal forecast model version to the next [17].

The fact that the stochastic physical parametrizations in System 4 improve the forecast quality of the tropical Pacific SSTs is in good agreement with the conclusions of Weisheimer *et al.* [13], who compared the impact of stochastic parametrizations in seasonal forecasts of tropical Pacific SSTs with the multi-model and perturbed physical parameter approaches to account for model uncertainty.

## 6. Quasi-stationary circulation regimes over the Pacific–North America region

The notion of how stochastic perturbations have the potential to impact on the long-term statistics of multi-modal systems has been discussed in the Introduction. While §§3 and 4 focused on analysing the impact of the operational stochastic physical parametrizations in ECMWF's seasonal forecasting system on the mean state and the statistics of the MJO, in this section, we shall discuss how the stochastic perturbations in System 4 affect the statistics of quasi-stationary circulation regimes over the PNA region. A previous study by Jung *et al.* [43] found that an earlier version of the backscatter scheme coupled to a cellular automaton, providing the spatial and temporal structure of the forcing, improved the frequency of occurrence of North Pacific weather regimes in atmospheric simulations with the ECMWF model driven by prescribed SSTs.

Atmospheric intraseasonal variability, especially in the extratropics during the cold season, is characterized by preferred large-scale flow patterns that appear repeatedly at certain geographical locations and persist beyond the typical lifetime of individual weather systems. These quasi-stationary flow patterns have non-Gaussian, or even non-modal, characteristics and are called weather regimes [44] or, more generally, circulation regimes [45]. Circulation regimes can be associated with significant temperature and precipitation anomalies [46,47]. Several observation-based and model studies have shown that ENSO forcing affects the relative frequency of occurrence of circulation regimes [45,48]. The ENSO forcing is particularly important for the PNA region, whose intraseasonal variability is directly linked to tropical Pacific SST anomalies [49,50].

The circulation clusters over the PNA region (140^°^E–80^°^W, 30^°^N–87.5^°^N) have been computed using daily data of geopotential height anomalies at 500 hPa during DJF for the re-forecast period 1981–2010 and applying the *k*-means clustering technique [50–53] in the phase space spanned by the first four EOFs to identify local density maxima. These EOFs explain about 50% of the total variance. We note that the spatial clustering patterns are very robust if the number of EOFs is increased. For the re-forecast simulations of System 4 and *stochphysOFF*, the November start dates and all 51 ensemble members were used in the computation of the regimes.

**Figure 9.**
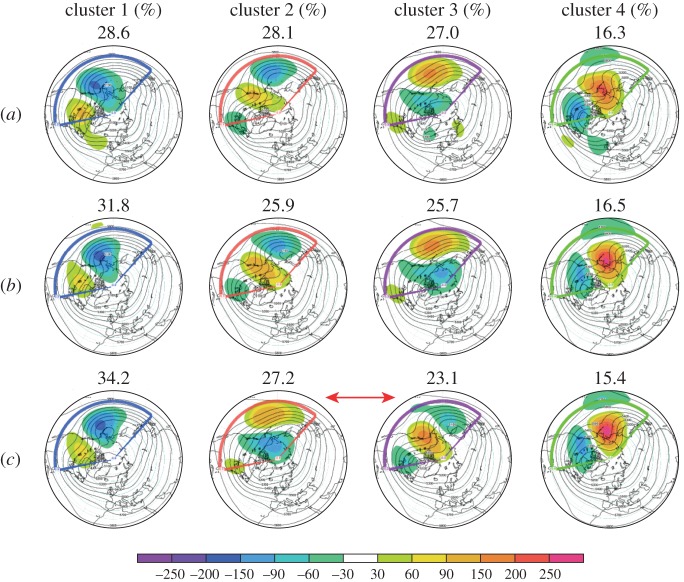
Circulation regimes over the PNA region in ERA-Interim reanalysis data (*a*), System 4 (*b*) and in *stochphysOFF* (*c*) for anomalies (shades) and full fields (contours) of Z500. The numbers indicate the relative frequency of occurrence of each cluster.

Composite maps of the four circulation regimes of ERA-Interim (*a*), System 4 (*b*) and *stochphysOFF* (*c*) are shown in figure 9. Cluster 1 of the reanalysis data is, in agreement with other studies [45,46,54,55], characterized by an eastward shift of the PNA pattern (‘Pacific trough’). The Pacific trough is the most populated cluster in the period considered. It occurs on 28.6% of the days. The 30 year time series of the frequency of occurrence of the Pacific trough cluster within each DJF season is highly positively correlated (*r*=0.7) with the observed multi-variate ENSO index [56] for the same period, indicating the strong link of the variability in the tropical Pacific on the formation of the Pacific trough flow regime. This is consistent with the results of Straus & Shukla [57], who described the mid-latitude Rossby wave response pattern to ENSO-driven anomalous tropical heat sources as a PNA-like pattern that is similar but not identical to the PNA pattern of Wallace & Gutzler [49].

Clusters 2 (28.1%) and 3 (27.0%) of ERA-Interim are the well-known positive and negative PNA patterns, respectively. They closely resemble opposite phases of the one-point correlation maps of Wallace & Gutzler [49]. The year-to-year variability in the frequency of occurrence of cluster 2 is only weakly related to the ENSO index (*r*=0.2). Cluster 3, the reverse PNA pattern, is negatively correlated (*r*=−0.5) with ENSO.

Cluster 4, the least populated cluster occurring on 16.3% of the days in ERA-Interim, is characterized by a blocked flow (Pacific blocking) and a pronounced ridge over Alaska, in agreement with the findings of Robertson & Ghil [46] and Straus *et al.* [45]. We find that this cluster is associated with cold ENSO events (*r*=−0.4).

The circulation regimes detected in System 4 and the *stochphysOFF* experiment are remarkably similar to those of ERA-Interim in the common period, indicating that the ECMWF seasonal forecasting system either with or without the stochastic parametrizations is able to reproduce the spatial structure of the flow regimes in the PNA sector. However, despite the good agreement between observed and simulated spatial patterns, there are in both sets of integrations non-negligible differences between the observed and the simulated regime frequencies. In particular, the average frequency of occurrence of cluster 1 (Pacific trough) is overestimated in both model runs: the estimates of 31.8% for System 4 and 34.2% for *stochphysOFF* correspond to a relative overestimation of 11% and 20%, respectively. Thus, the stochastic parametrizations in System 4 help to reduce the strong overestimation of cluster 1.

In contrast to the strong overestimation of the Pacific trough regime by the model, both System 4 (25.9%) and the *stochphysOFF* experiment (23.1%) underestimate the occurrence of the positive PNA regime in cluster 2. The degree of underestimation is by 8% of the ERA-Interim frequency of 28.1% in System 4 and by a substantial 18% relative to ERA-Interim for *stochphysOFF*.

The negative PNA regime frequency of cluster 3 in the reanalysis is underestimated by System 4 (25.7% absolute frequency of occurrence) and slightly overestimated by *stochphysOFF* (27.2%). The underestimation of the positive PNA regime along with the overestimation of the negative PNA regime for the *stochphysOFF* simulations result in a change of the order of the four circulation regimes when sorted according to their frequencies of occurrence for *stochphysOFF* (figure 9).

As in ERA-Interim, cluster 4 represents the Alaskan Ridge pattern in all sets of integrations. However, the experiment without representation of model error *stochphysOFF* (15.4%) underestimates the number of days of such a blocked flow regime when compared with ERA-Interim by a relative difference of 6%. System 4 (16.5%) marginally overestimates the occurrence of the Alaskan Ridge cluster by 1%.

The atmospheric flow regimes over the North Pacific area are sensitive to the state of the tropical Pacific Ocean. Our results show that when the stochastic physical parametrizations in the atmosphere are switched off, the two most populated regimes (Pacific trough and negative PNA) are also the two regimes with the strongest correlation to the ENSO forcing (*r*=0.7 and −0.5, respectively). The overpopulation of these two ENSO-related regimes in *stochphysOFF* indicates that the ECMWF coupled system is too sensitive to the boundary forcing in the Equatorial Pacific. Interpreting these results with our conceptual understanding of the impact of stochastic perturbations in a multiple potential well environment, as discussed in the Introduction, implies that the atmospheric state vector without these perturbations tends to prefer the quasi-stationary circulation regimes associated with the strongest ENSO forcing. As a consequence of this, the frequency of occurrence of the most ENSO-sensitive regimes is overestimated, whereas the frequency of the regimes less responsive to the forcing is underestimated.

Adding stochastic perturbations to the system, as in System 4, can trigger regime transitions of the state vector from the absolute minimum of the multiple potential well to further local minimum quasi-stationary states that were otherwise populated less frequently [16]. This is indeed what happens in System 4. The impact of the stochastic parametrizations on the North Pacific circulation regimes is such that they tend to reduce the frequency of occurrence of those two clusters in *stochphysOFF* that have the strongest relationship to ENSO forced variability, the hugely overpopulated Pacific trough and the slight overestimated negative PNA cluster. At the same time, the stochastic parametrizations in System 4 increase the frequency of occurrence of the otherwise strongly under-represented positive PNA regime.

It is interesting to note that while the excessive sensitivity of the ECMWF coupled system to the equatorial Pacific SSTs does affect the circulation regime frequencies of occurrence, it does not affect the regime structure *per se*. The four circulation patterns are very well reproduced in both the *stochphysOFF* and System 4 simulations. This might be further evidence for the suggested paradigm that the time-mean response of a system to some imposed forcing manifests itself through a change in frequency of their naturally occurring quasi-stationary regimes [48,58,59].

A similar analysis over the Euro-Atlantic area was performed but no evidence for differences between System 4 and *stochphysOFF* in terms of circulation regime structure or frequency of occurrence could be found. It has to be noted that the Euro-Atlantic area is a very noisy region in the model (signal-to-noise ratio of approx. 0.2), and thus it is intrinsically more difficult to detect any impact of stochastic perturbations.

## 7. Summary and conclusion

The impact of two stochastic parametrization schemes in the atmosphere of the ECMWF coupled seasonal forecast System 4 has been analysed. The schemes which are also used in ECMWF's medium-range ensemble forecasts perturb the total tendencies of all diabatic (parametrized) processes (SPPT scheme) and the backscatter of kinetic energy from small to large scales (SPBS scheme). The impact has been quantified by comparing a 30 year retrospective forecast series with lead times of two to four months with and without these two schemes activated. It was found that the system without stochastic perturbations generates large areas of excessively strong tropical convection, especially over the Indonesian warm pool area and the tropical Western Pacific. The stochastic schemes, and in particular SPPT, weaken the convective activity in these areas, leading to reduced biases of OLR, cloud cover, precipitation and near-surface wind. It should be noted, however, that the stochastic schemes do not eliminate these biases entirely. The stochastic backscatter scheme has an overall neutral impact.

A long-standing problem in modelling tropical convection is the Madden–Julian intraseasonal oscillation. The ECMWF seasonal forecast system generally underestimates the frequency and amplitude of daily MJO events. It has been demonstrated that the stochastic parametrization schemes help to improve the MJO statistics consistently across start dates throughout the year. However, the magnitude of frequency improvements of approximately 10% is rather small compared with the overall underestimation of approximately 30%. The largest contribution to the improvements originates from the SPPT scheme. The perturbed physical tendencies also have a positive impact on the distribution of the amplitudes of MJO events by increasing the number of stronger MJO events and reducing the number of weaker events.

Along with systematic improvements in the tropical climate, it was found that ENSO forecast quality, in particular over the tropical Western Pacific, has increased in System 4 owing to the stochastic physics scheme. After forecast month 1, the schemes led to a systematic and significant reduction of the ensemble mean RMSE and a substantial increase in the ensemble spread of SST forecasts in the Niño4 region as well as to increased anomaly correlations. Again, the SPPT scheme has the largest contribution to these improvements, whereas the SPBS scheme shows little impact on seasonal time scales.

Finally, a cluster analysis of the mid-latitude quasi-stationary circulation regimes over the North Pacific and North American region was performed. The impact of the stochastic parametrizations on the regimes is such that they tend to reduce the frequency of occurrence of those two clusters in *stochphysOFF* that are strongest linked to ENSO forced variability (Pacific trough and the negative PNA cluster). At the same time, the stochastic parametrizations in System 4 increase the frequency of occurrence of the otherwise strongly under-represented positive PNA regime. This shift in the frequency of occurrence of circulation regimes owing to stochastic perturbations is in agreement with the notion of noise-activated regime transitions.

What are the implications of this study? The stochastic parametrization schemes used in this model were primarily developed for application in NWP. Indeed, they have been successfully used in ECMWF's operational medium-range (10–15 days) ensemble forecasts for some years. More recently, the SPPT scheme has also become part of the ensemble of the data assimilation system at ECMWF to provide ensembles of initial conditions [60]. The successful operation of these schemes on such a range of time scales from a few hours to several months can be seen as an exemplification of the seamless prediction concept where climate models should be tested in weather prediction mode where more verification data are available. It suggests that the results presented here may also be relevant on longer multi-decadal time scales and that stochastic parametrizations should now be developed for multi-decadal climate predictions using Earth-system models.

A question that remains open is why the stochastic backscatter parametrization has so little effect on the seasonal forecast simulation. Finding the reasons behind this will be a focus of future investigations. One possible explanation could be that the model error component owing to the upscale energy transfer is relatively small and that the total model error is dominated by contributions from those physical sub-grid-scale parametrization schemes that are perturbed in the SPPT scheme, for example cloud microphysics and convection. A further uncertain effect is the impact of the temporal perturbation time scales of 6 h, 3 and 30 days used in SPPT but not in SPBS. Here, the longest scale projects well onto the seasonal time scale of the simulations discussed in this paper. However, initial results from a sensitivity study with varying perturbation amplitudes for the three time scales showed no clear indication of a systematic effect.

For the future, it is planned to investigate in more detail how and why the stochastic parametrization scheme influences tropical convection. First indications of daily grid-point precipitation time series point towards a potential forced suppression of convective rainfall and thus an increase in dry days (reduction of drizzle days) when the stochastic perturbations are applied.
